# ^1^H-NMR spectroscopy metabonomics of reactive, ovarian carcinoma and hepatocellular carcinoma ascites

**DOI:** 10.1515/pp-2020-0113

**Published:** 2020-05-12

**Authors:** Lucio Zennaro, Lorenzo Nicolè, Paola Vanzani, Filippo Cappello, Ambrogio Fassina

**Affiliations:** Department of Molecular Medicine, University of Padova School of Medicine and Surgery, Padova, Italy; Department of Medicine, University of Padova School of Medicine and Surgery, Padova, Italy; Department of Medicine, University of Padova School of Medicine and Surgery, Padova, Italy

**Keywords:** cancer markers, hepatocellular carcinoma, metabonomics, ovarian carcinoma, peritoneal effusion

## Abstract

**Background:**

Metabolomic profiling of human malignant effusion remain a field poorly investigated. Proton nuclear magnetic resonance (^1^H-NMR) spectroscopy is a rapid relatively low cost technique, and effusion is an optimal biospecimen suitable for metabonomic investigations. With this study we addressed metabolomic profiling of malignant ascitic effusion (mAE) from patients with high grade serous ovarian carcinoma (HGSOC), Hepatocellular carcinoma (HCC), and benign AEs (bAEs) from patients with reactive peritonitis.

**Methods:**

Metabolic profiling with ^1^H-NMR was performed on 72 AEs (31 HGSOC, 16 HCC and 25 bAE) prospectively collected in our cytology service. Histological confirmation was requested for all malignant case. Multivariate analysis comprising PCA and PLS-DA was applied to discover metabolites suitable to differentiate effusions among the investigated groups.

**Results:**

^1^H-NMR metabonomic analysis showed clearly different spectra for malignant and benign AEs, as well as for HGSOC vs. HCC effusion. When compared with HCC effusions, the HGSOC effusion were enriched, among all, in alanine, lipids, N-acetyl groups and phenylalanine and depleted in glutamine.

**Conclusions:**

Subject to validation in further larger studies, ^1^H-NMR metabonomics could be an effective and reliable ancillary tool for AE investigations and diagnosis particularly in acellular effusions.

## Introduction

Ascitic effusion (AE) is defined as the presence of abnormal fluid quantity within the peritoneal cavity. The most common cause of ascites is portal hypertension and hypoproteinemia resulting from cirrhosis, but other frequent causes include malignancies (e. g. ovarian, hepatic or gastrointestinal cancers), heart failure [1, 2].

When the effusion is symptomatic, abdominal paracentesis is required to give immediate relief to the patient, also aimed to collect the fluid for diagnosis and research. The initial evaluation of AE relies upon the assessment of the effusion appearance, determination of serum-to-ascites albumin gradient (SAAG), white blood cell count, and determination of total protein concentration [3, 4]. SAAG is the most used criterion to differentiate transudative AE, (SAAG>1.1, mainly related to portal hypertension) from exudative AE [1, 5]. Additional evaluations can be necessary for the differential diagnosis, including microbiological exams, biochemical analysis and cytology. In particular, cytology, allowing the direct recognition of malignant cells and their characterization, is considered the gold standard for the diagnosis of malignancy in AE (mAE) [5]. However, sensitivity of cytology in AE is strongly affected by the underlying disease, since several tumor arising within or close to the peritoneum do not spread cells within the peritoneal cavity resulting in negative cytology, such as hepatocellular carcinoma (HCC) [6, 7].

Determination of various tumor markers, such as carcinoembryonic antigen (CEA), cancer antigen (CA) 125 and CA19.9, has been proposed as an additional method to increase the sensitivity and accuracy of mAE diagnosis [8, 9, 10, 11]. However, since the precise value of this approach remains unclear and largely variable among different laboratories, in clinical practice tumor markers measurement is rarely used in effusion.

Moreover, diagnostic role of molecular assays based on the isolation of nucleic acids (DNA or microRNA), has shown promising results; nonetheless this approach is still in its infancy and published data are so far inconsistent [12, 13].

More recently, metabonomics by high resolution ^1^H-NMR spectroscopy has proved useful in effusion analysis. This technique allows the metabolic profiling of tissues and biologic fluids. ^1^H-NMR spectroscopy can be used for the detection of metabolic alterations associated with the presence of pathologic processes, and metabolites could be recognized as potential biomarkers [14, 15, 16]. The vast majority of clinical research by ^1^H-NMR spectroscopy has usually been carried out on urine and serum specimens [17], since such samples can be withdrawn by relatively non-invasive means and perhaps because these fluids have the greatest potential to provide immediate diagnostic information [18, 19]. There are very few papers on AE based on ^1^H-NMR metabonomics analysis, although some recent works confirm its relevance [20, 21]. When compared with urine and plasma, effusion have the advantage to derive from a closed cavity lined by serous membranes that act as selective semi-permeable filter with high selective capacity in filtrating and concentrating small plasma metabolites. For these reasons, effusion is an ideal fluid to investigate the presence of small molecules, and hopefully new cancer biomarkers, less masked here from the large proteins (albumin) present at high concentrations in the blood [22]. Within this context, the high sensitivity of the ^1^H-NMR spectroscopy renders effusion particularly suitable to research new tumor markers, and in this work we assessed the contribution of ^1^H-NMR spectroscopy as reliable diagnostic tool for classify mAE vs. bAE also testing the possibility of discriminating specific malignancies as HGSOC and HCC. The validation of the results obtained was performed by comparison with traditional cytology analysis and histological confirmation in cases where effusions were typically negative for malignant cells (i. e HCC). Multivariate statistics (PCA, PLS-DA) was then applied to spectroscopic data in order to find a set of metabolites able to drive effusion discrimination and that could be useful as cancer biomarkers.

## Materials and methods

### Human peritoneal effusion harvesting

Seventy two AEs were prospectively collected between 2018 and 2019 at the Surgical Pathology and Cytopathology Unit at Padua University, 31 patients had HGSOC, 16 HCC and 25 AEs derived from patients with reactive peritonitis (clinical and pathological data are reported in Table 1).

**Table 1:** Basic clinical data of patients included in HGSOC, HCC and control cohort.

	OC- AE	HCC- AE	Reactive AE
Total sample, n	27	16	25
Female, n	27	1	21
Male, n	0	15	4
Age, range, y	47-85	45-75	34-51
Age, mean ± st dev. y	68.3 ± 10.4	61.5 ± 7.5	57.8 ± 20.8
Age, median, y	67	62.5	61

HGSOC, high grade serous ovarian carcinoma; HCC, hepatocellular carcinoma; AE, ascitic effusion.

All the AE samples were centrifuged within 1 h after withdrawal at 2000×*g* for 10 min at room temperature. The precipitate was used to prepare two slides for cytology, while 4 mL of each supernatant were divided in two parts (to produce two identical samples of the same AE, for repeated analysis), transferred in 2 mL sterile tubes, and centrifuged at 2000×*g* for 20 min at 4 °C. The AE supernatants were then stored at −80 °C until use.

**Figure 1:**
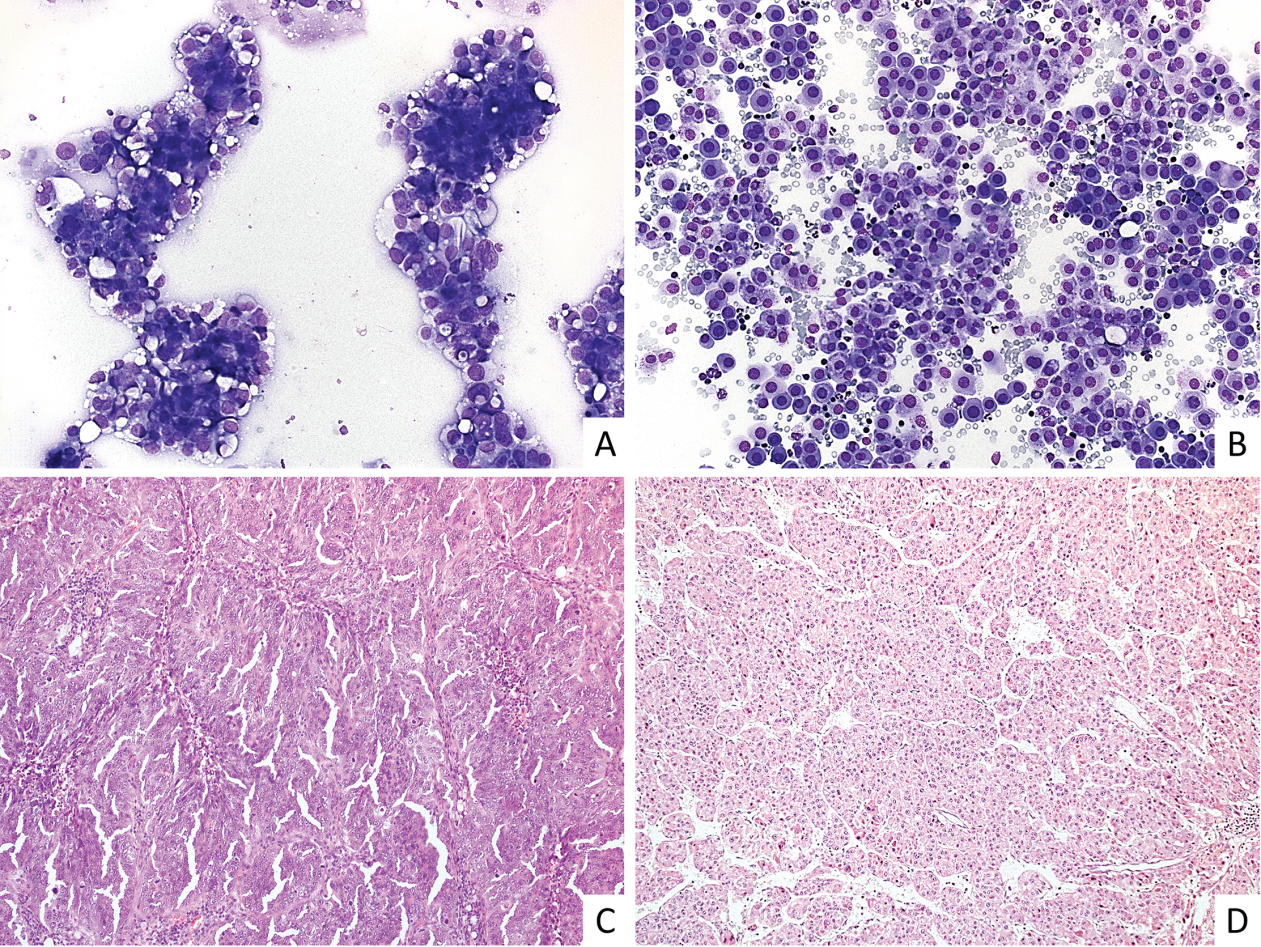
Representative images of a positive effusion from a patient included into the HGSOC cohort (A) and a negative effusion from a patient with HCC (B). Corresponding histology is shown in (C) for HGSOC and in (D) for HCC.

Two cytopathologists (LN and FC) re-examined all cases independently and defined the three cohorts of HGSOC, HCC and bAE. Diagnosis was confirmed considering effusion morphology, ancillary test as immunohistochemistry on cell block where feasible, and considering clinical history. In the HGSOC and HCC cohorts we included only cases with unequivocal history of malignancy provided by histological confirmation; patients were enrolled regardless the percentage of tumor cells, since we focused on liquid composition of confirmed neoplastic effusions (Figure 1).

(A and B) May-Grunwald Giemsa stain, original magnification x200. (C and D) Hematoxylin and Eosin stain, original magnification x200.

### Chemicals and reagents

All chemicals were purchased from Sigma-Aldrich: Na_2_HPO_4_ anhydrous and NaH_2_PO_4_ anhydrous (≥99%), sodium azide (NaN_3_), D_2_O 99.98%, 2,2,3,3-*d4*-3-(trimethylsilyl) propionic-acid sodium salt (TSP-*d4*, 98%D atom) and methanol-*d4* 99.8%. TSP-*d4* was used as internal chemical shift reference for NMR spectra, while methanol-*d4* was used for NMR temperature calibration.

### Buffer for NMR analysis

Phosphate buffer NaPi was prepared in H_2_O:D_2_O (1:1) to a final concentration of 1.5 M, pH 7.4. TSP-*d4* and NaN_3_ (5 and 15 mM final concentration, respectively) were added to the buffer solution as internal chemical shift reference (TSP-*d4*) and for antibacterial purposes (NaN_3_). All the AE samples were diluted in this buffer after their de-freezing, immediately before the NMR analysis.

### AE sample treatment for NMR investigation

We followed a rigorous protocol for NMR sample preparation. To minimize the variability in pH that potentially characterize the ascitic effusions from different individuals, a AE sample was defrosted from −80 °C immediately before the NMR analysis, quickly centrifuged at 12,000×g for 5 min at 4 °C and rapidly diluted in phosphate buffer (80:20 V/V), final pH 7.30 ± 0.02. Finally, 600 μL of the buffered sample are transferred into 5 mm NMR tube and immediately inserted in the spectrometer for the metabonomic assay.

### NMR equipment and ^1^H-NMR data acquisition

^1^H-NMR spectra were acquired using a Bruker Advance III spectrometer (Bruker Biospin, Rheinstetten, Germany) operating at 300.13 MHz proton Larmor frequency. A 5 mm outer diameter ^1^H – ^13^C probe was used, equipped with Z gradient facility. For each sample three different one-dimensional (1D) NMR spectra were acquired, namely 1D Noesy sequence (“noesygppr1d” in Bruker library), 1D Carr–Purcell–Meiboom–Gill (CPMG) spin-echo sequence, (“cpmgpr” in Bruker library), and 1D Diffusion-edited sequence (“ledbpgppr2s1d” in Bruker library). The 1D Noesy sequence was used to collect each ^1^H-NMR spectrum as it is, without applying any digital filter that generally is used to attenuate or enhance the signals from low or high molecular weight species, thus allowing a more reliable determination of the metabolites’ concentrations. The water peak suppression was obtained by pre-saturation using a standard zgpr pulse sequence. The 1D CPMG sequence was used to attenuate the broad signal from macromolecules (e. g. proteins) that may be present in the sample, thus permitting a better evaluation of low molecular weight species (metabolites). This sequence was preferred to de-proteinization procedures, in order to minimally perturbate the biological fluid [20]. As for the NOESY sequence, for CPMG sequence the water peak suppression was also obtained by pre-saturation using a standard zgpr pulse sequence. We used the 1D Diffusion-edited sequence to better visualize the signal from higher molecular weight species (e. g. lipids, lipoproteins). The spectra were recorded with 64 or 2048 scans, 64 K data points, a spectral width of 30 ppm (8971 Hz), 4 dummy scan and a relaxation delay of 4 s. All 1D spectra were processed with line broadening of 0.3 Hz before the Fourier Transform. Spectra acquisition and processing were both performed by using TopSpin 3.6.1 software (Bruker Biospin). The chemical shifts were internally calibrated to the TSP peak at 0.0 ppm. All NMR experiments were acquired at the constant temperature of 310 K, with temperature stabilization of approximately 0.1 K. For temperature equilibration, the samples were kept for 5 min inside the NMR probe head before starting the spectrum acquisition. The assignments of various resonances were made using metabolite NMR databases (BBIO-REFCODE 2 database, Bruker Biospin), Human Metabolome Database HMBD (www.hmdba.ca) and literature data based on chemical shifts and split patterns of proton signals [23].

### Multivariate statistical analysis

A total of 72 spectra, 31 from HGSOC, 16 from HCC and 25 from control patients, have been processed with multivariate analysis. The PCA followed by the PLS-DA were performed by AMIX 4.0.1 software package (Bruker Biospin). Each 1D spectrum area was bucketed into frequency windows of 0.05 ppm (rectangular bucketing amplitude) in the range between 0.50 and 10.00 ppm. The region corresponding to water (4.50–5.00 ppm) was excluded during bucketing to avoid artifacts due to the pre-saturation of water. The spectral content of each bucket was converted to the numerical value corresponding to the area subtended by the portion of the spectrum enclosed in the bucket. Thus, each bucket represents the original variable to which the statistical analysis is applied. All original variables were preprocessed with mean-centered and Pareto-scaling procedures before being evaluated by the multivariate statistical analysis. As first step, the PCA analysis was performed to obtain the principal components of the variance (PC). Principal component (PCi) is a linear combination of all the original variables, and each successive PCi (progressively at a lower extent) describes the maximum amount of variance possible in the data set. PCA was used to find if the spectra could be clustered in separate groups containing similar spectra. Based on these PCA results, PLS-DA was performed to assess the strength of such a separation, using the condition control/cancer (either HGSOC or HCC, depending on the case) as classifier for the clustering.

## Results

### Metabolites spectra

**Figure 2:**
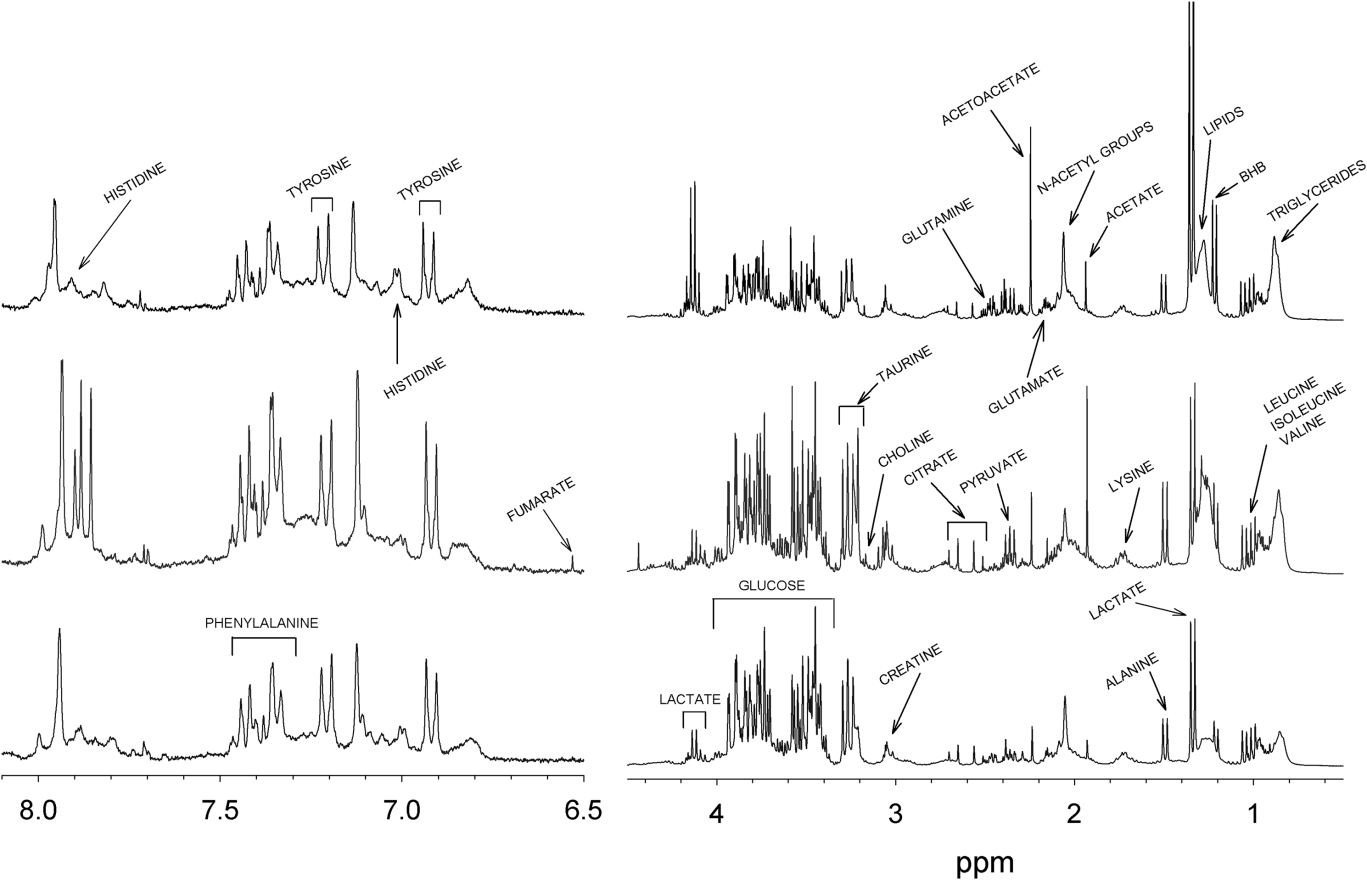
Typical ^1^H-NMR spectra of AE from a patient with HGSOC (top), from a patient with HCC (middle) and from a patient of the control group (bottom).

Figure 2 shows typical ^1^H-NMR 1D CPMG spectra of AE from a patient with HGSOC (top), a patient with HCC (middle) and from a patient from the Control group (bottom). The metabolites identifiable in the above mentioned spectra are listed in Table 2 and were found to give signals in four spectral regions: (1) the region from 0.8 to 1.80 ppm: lipids, triglycerides, branched-chain amino acids (leucine, isoleucine and valine), aliphatic amino acids (alanine, lysine), organic acids (lactate, butyrate) and β-hydroxybutyrate (BHB); (2) the region from 2.0 to 2.50 ppm: other aliphatic amino acids (glutamine, glutamate), other organic acids (pyruvate, citrate), other ketone bodies (acetoacetate) and N-acetyl groups; (3) the region from 3.0 to 4.0 ppm: sugar (glucose) and alcohol (glycerol), creatine, creatinine, taurine and choline; (4) the region from 6.90 to 7.50 ppm: aromatic amino acids (tyrosine, histidine, phenylalanine).

**Table 2:** Metabolites identified by the ^1^H-NMR metabonomic assay.

Metabolites	Chemical shifts (ppm)	HCC	OC
Triglycerides	0,80–0,90	↑	↑
Amino acids (leucine, isoleucine, valine)	0,94–1,08	↑	↑
Alanine	1,45		↑
β-Hydroxybutyrate	1,22; 2,32; 2,43	↑	↑
Butyrate	0,88; 1,55; 2,14		
	1,22–1,31		↑
Lipids	5,3		
	1,35	↓	↑
Lactate	4,08–4,18	↓	↑
Lysine	1,7	↑	↑
Acetate	1,95	↑	↑
Glutamate	2,06–2,14		
N-acetyl groups	2,05		↑
Acetoacetate	2,24	↑	↑
Pyruvate	2,38	↑	↑
Glutamine	2,40–2,45		↓
Citrate	2,52–2,72		
Creatinine – creatine	3,06; 3,95		
Choline	3,20	↑	↑
Taurine	3,27		
Glucose	3,38–3,90	↑	↓
ɑ-glucose	5,25	↑	↓
Glycerol	3,58; 3,65	↑	↓
Fumaric acid	6,58		
Tyrosine	6,93; 7,2		
Histidine	7,15; 7,95		
Phenylalanine	7,33; 7,38; 7,43		↑
Formate	8,48		

The signals positions are reported as chemical shift with respect to the TSP signal, used as reference at 0 ppm. The increase (up arrow) or decrease (down arrow) in concentration of the metabolites in mAE (HGSOC and HCC) is referred to the control group (bAE). Where no arrow is present, the metabolite concentration is statistically invariant between mAE and bAE.

### Multivariate statistics

The results from the PCA analysis of the data embedded in the ^1^H-NMR spectra were visualized in graphical mode, by plotting the PC scores and PC loadings for any PCi–PCj pair: each point in the scores plot represents an individual patient (i. e. an individual NMR spectrum), and each point in the corresponding loadings plot represents one spectral region. The points that in the loadings plot move away from the center (coordinate 0,0) represent the spectral regions that more strongly influence patterns in the scores plot and, consequently, the molecules that mostly influence patterns in the NMR spectra, or, in other words, the molecules that mostly influence the clustering of NMR spectra/patients in separate groups. The reliability of the qualitative evaluation of the clustering highlighted by the graphical presentation of the results is given by the percentage of the variance explained by the two PCi to which the plots refer: the higher the percentage of total variance explained, the higher the reliability of the clustering. Performing a PLS-DA analysis on the same data set as PCA can give further support to the reliability of the separation or weaken it: if the clustering shown by PCA is actually reliable, the PLS-DA analysis must confirm it, with a separation between groups that is generally at least equal if not better. On the contrary, a poor clustering getting out from PLS-DA makes debatable the validity of the separation obtained with the PCA.

### HGSOC vs. control group

**Figure 3:**
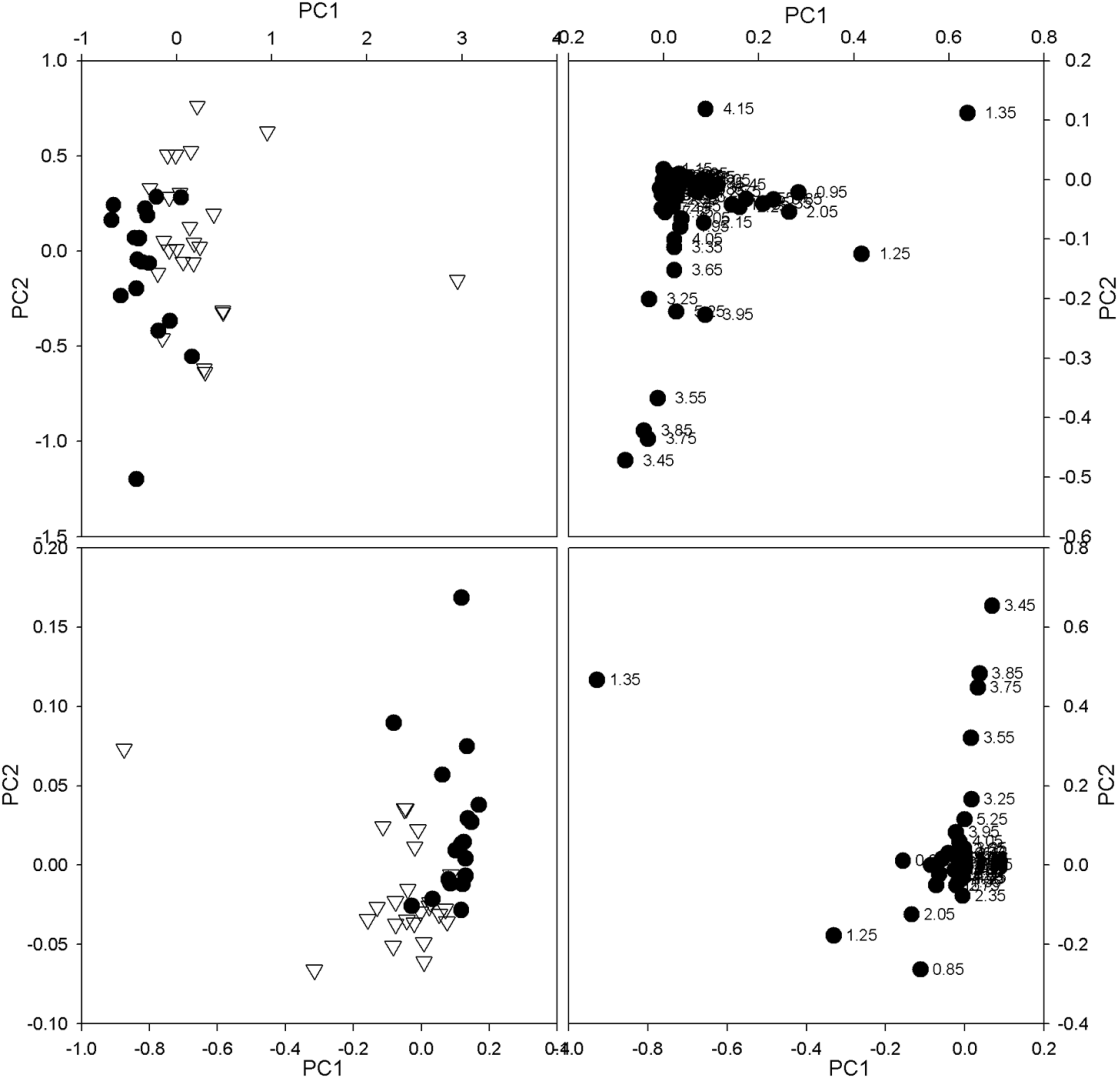
Top: scores plot (left) and loadings plot (right) obtained from PCA performed on m-AEs from HGSOC patients and from control group. Bottom: scores plot (left) and loadings plot (right) obtained from PLS-DA performed on the same dataset as PCA.

In Figure 3 (top) are shown the scores and the loadings plots of the PCA performed on HGSOC and Control groups. The plotted scores indicate a clear clustering of the spectra, with PC1 and PC2 explaining 65.38% of the total variance (45.76% and 19.62%, respectively). The spectral regions that in the correlated loadings plot appear to be responsible for such a separation are those which contains the signals from lipids, triglycerides, lactate, leucine, isoleucine, valine, alanine, lysine, phenylalanine, β-hydroxybutyrate (BHB), acetate, acetoacetate, N-acetyl groups, pyruvate, taurine, choline, glutamine and glycerol. Figure 3 (bottom) shows the scores and the loadings plots resulting from the PLS-DA analysis performed on the same dataset as PCA, using as classifier for the Discriminant Analysis (DA) the condition control vs. cancer (with 0 assigned to control condition and 1 assigned to ovarian carcinoma condition). The graphical separation getting out the PLS-DA was as clear as that obtained by PCA, thus confirming the reliability of the latter. The low value of 0.174 obtained for the root mean square error of calibration (RMSEC) of the PLS-DA confirmed on a numerical basis the good clustering graphically observed (the lower RMSEC, the more reliable the clustering).

In the scores plots triangles refer to m-HGSOC-AEs, filled circles to bAE**.**

### HCC vs. control group

**Figure 4:**
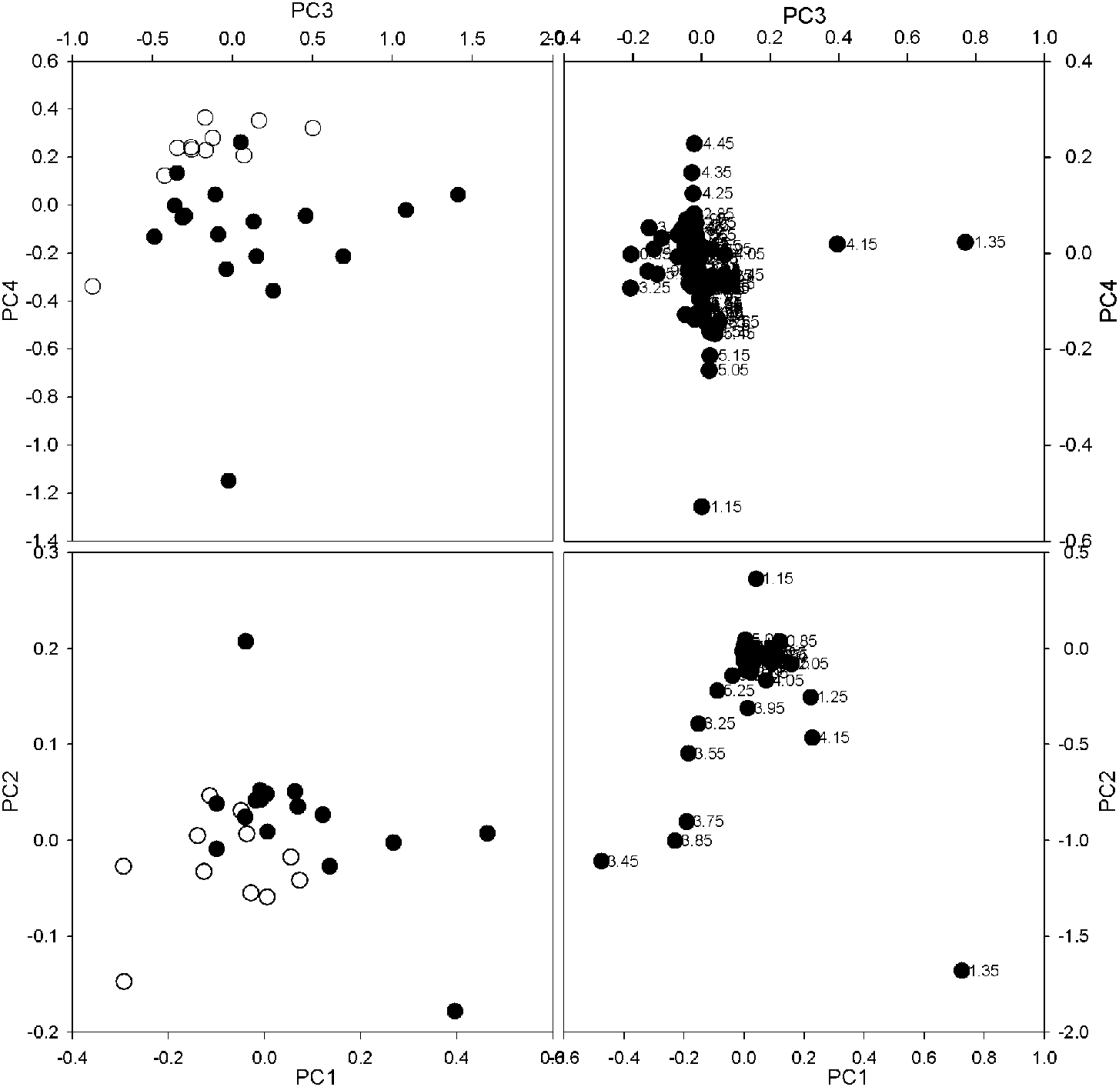
Top: scores plot (left) and loadings plot (right) obtained from PCA performed on m- AEs from HCC patients and b-AEs from control group. Bottom: scores plot (left) and loadings plot (right) obtained from PLS-DA performed on the same dataset as PCA.

The same statistical approach was used to evaluate the metabolic information embedded in the ^1^H-NMR spectra of ascitic effusions collected from HCC patients. The scores and the loadings plots of the PCA performed on HCC and Control groups are shown in Figure 4 (top). The scores plot shows a weaker, but still clear separation of the spectra in two groups, with PC3 and PC4 explaining 20.31% of the total variance (14.41% and 5.90%, respectively). The spectral regions responsible for such separation in the correlated loadings contain signals from triglycerides, leucine, isoleucine, valine, lactate, lysine, phenylalanine, β-hydroxybutyrate (BHB), acetate, acetoacetate, pyruvate, taurine, choline and glycerol.

In the score plots outlined circles refer to m-HCC-AEs, filled circles to bAE.

The reliability of the clustering found by PCA on HCC vs. Control group was indeed confirmed by the PLS-DA analysis performed on the same data set: the scores and loadings plots calculated by PLS-DA (Figure 4, bottom) showed a good separation of the NMR spectra in two groups, with a RMSEC value (0.157) as good as that found for HGSOC vs. Control group.

### HGSOC vs. HCC

**Figure 5:**
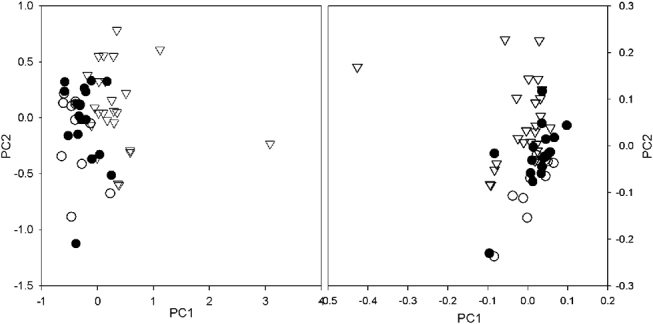
Scores plot obtained from PCA (left) and PLS-DA (right) performed on the entire ensemble of AEs.

To assess the ability of ^1^H-NMR metabonomics to discriminate a mAE (HGSOC, HCC) from controls (bAE), and to recognize specific malignancies (e. g. HGSOC vs HCC), we submitted the entire ensemble of recorded spectra to PCA and PLS-DA analysis. The choice of considering all the ascites together and not simply HGSOC vs. HCC, was driven by the fact that the separation from Controls is stronger for HGSOC than for HCC, and there is the risk of finding an artificial discrimination between the two specific cancers due essentially to the weak separation between HCC and controls, and therefore due to the statistical similarity of Controls and HCC with respect to HGSOC. Figure 5 shows the scores plot obtained by PCA (left) and PLS-DA (right) performed on the entire ensemble of spectra. The clear clustering shown by the PCA result (with PC1 and PC2 explaining 65.60% of the total variance) highlights the presence of three groups of spectra. The reliability of the clustering is confirmed by the good graphical separation shown by the PLS-DA. Moreover, having used as classifier for the Discriminant Analysis the triple condition Control – Cancer A – Cancer B (with 0 assigned to control condition, 1 assigned to HGSOC condition and 2 assigned to HCC condition), the PLS-DA confirmed the presence of three different classes of AE.

Triangles: m-HGSOC-AEs, outlined circles: m-HCC-AEs, filledcircles: bAEs).

## Discussion

Histology remains the benchmark of diagnosis in cancer, but biopsy or surgery is invasive, unsuitable for early diagnosis and frequently not practicable in patients with poor performance score. Thus, the development of less invasive and more cost-effective diagnostic procedures is a priority for clinical researchers. Within the clinical management of patients with peritoneal effusion, cytology is the most diffuse diagnostic test. However, its reliability may be hindered by the overlapping features of benign and malignant proliferations, with a concrete risk of false results. Moreover, several malignancies do not spread cells within the effusion, resulting in a false-negative cytology. The introduction of immunocytochemical reactions on cell blocks has increased the diagnostic performance, although still unsatisfactory due to cell paucity. Proteomic and genomic profiling have proved to be useful tools for cancer diagnosis, but require a significant number of tumor cells for a reliable diagnosis, with so far only few molecular tests validated and approved for clinical use [12].

In this scenario ^1^H-NMR based metabonomics could offer the possible finding of a set of biomolecules potentially interesting as cancer biomarkers [24, 25].

Our ^1^H-NMR results highlighted that, among all the metabolites identified (Table 2), the differentiation between HCC, HGSOC and bAE can be related only to some of them. In particular, the major contribution to the clustering of HGSOC and bAE in two separate groups is due to the increase of the signal of lipids, triglycerides, lactate, the branched-chain amino acids (leucine, isoleucine and valine), some other amino acids (alanine, lysine, phenylalanine), β-hydroxybutyrate (BHB), acetate, acetoacetate, N-acetyl groups, pyruvate, taurine/choline, and to the decrease of glutamine and glycerol signals (Table 2). On the contrary, glutamate, some aromatic amino acids (tyrosine, histidine), butyrate, citrate, creatinine, creatine, fumaric acid and formate signals are statistically negligible for HGSOC and bAE. In the differential between HCC and bAE, the major contribution to the clustering is due to the increase of triglycerides, β-hydroxybutyrate, glycerol, the branched-chain amino acids (leucine, isoleucine and valine), lysine, acetate, acetoacetate, pyruvate, taurine and choline, and to the decrease of the only signals given by lactate. All the other signals identified in the NMR spectra of the HCC were found to be statistically non-significant with respect to the control group. It should be noted that, unlike HGSOC, where PC1 and PC2 were considered, for HCC we used PC3 and PC4. In fact, even if the percentage of the total variance by PC1 and PC2 was higher (64.90%, with 38.66% explained by PC1 and 26.24% explained by PC2) than that explained by PC3 and PC4, the graphical separation obtained in the space PC1 vs. PC2 was not as clear as that shown in the space PC3 vs. PC4. This latter indicates that metabolic variations with high variance have a low discriminant impact in differentiating the HCC from the bAE group. Conversely, other metabolic variations with individual small effect on variance showed if considered globally a good discriminant effect between HCC and the bAE group.

The results obtained indicate that the differentiation of the HGSOC metabolic profile vs. bAE is due to a range of metabolites wider than in HCC. However, this could be related to the low number of our HCC which could not guarantee a sufficiently robust statistical separation from the control group, and deserves to be further verified on a greater number of cases. Since our HGSOC and HCC were compared with the same control group, it could be argued that, among the metabolites responsible for the separation of mAE vs. bAEs, those that vary only for a specific mAE could be labeled as specific and diagnostic. If so, then lipids, glutamine, alanine, phenylalanine and N-acetyl groups should be specific for HGSOC vs. HCC. Further comparing HGSOC and HCC, a particular consideration seems to deserve lactate and glycerol, as both have opposite variations in the two cancer types compared to control group: lactate increases and glycerol decreases in HGSOC, the opposite is in HCC. Moreover, it may be noteworthy that the metabonomics statistical approach indicates that the signals from glucose decrease in HGSOC and increase in HCC from controls. This, when associated with lactate variations (increase in HGSOC, decrease in HCC), would suggest an important difference in balance of the glycolytic metabolic pathway in the two cancer histotypes, with the HGSOC characterized by a Warburg effect not observed in HCC [26].

In conclusion, our study indicates that ^1^H-NMR metabonomics is a simple and reliable technique to distinguish between mAEs and bAEs. And in more detail, analysis may differentiate HGSOC-derived effusion from HCC-derived effusion. Although such results are promising, we must point out that our data should be validated in larger cohorts of patients, and that appropriate statistical models should be setup to create and test the accuracy of a classifier based on the metabonomics profiling.
